# School climate’s effect on hospitality department students’ aesthetic experience, professional identity and innovative behavior

**DOI:** 10.3389/fpsyg.2022.1059572

**Published:** 2022-12-05

**Authors:** Weixin Lin, Yuan-Cheng Chang

**Affiliations:** ^1^Department of Visual Communication Design, Hainan Vocational University of Science and Technology, Haikou, Hainan, China; ^2^Department of Education Management, Chinese International College, Dhurakij Pundit University, Bangkok, Thailand

**Keywords:** school climate, aesthetic experience, professional identity, innovative behavior, hospitality department students

## Abstract

This study investigated the effects of school climate and students’ aesthetic experience on their professional identity and innovative behavior. A survey was conducted with 385 students from hospitality-related departments of colleges and universities in Hainan, China, and the data were analyzed using a hierarchical linear model (HLM). Using the criteria constituting the students’ aesthetic experience scale proposed by Chang, it was found that teacher support can improve students’ professional identity; school climate and students’ understanding of beauty and full experience contribute to the development of students’ innovative behavior; students’ understanding of beauty and full experience have mediating effects between teacher support and professional cognition; students’ understanding of beauty and full experience have mediating effects between student support and innovative behavior; student support positively moderates the relationships between full experience with professional cognition and students’ appraisal of the hospitality industry; and teacher support positively moderates the relationship between students’ full experience and professional emotion. Therefore, teacher support under school climate and students’ understanding of beauty and full experience under aesthetic experience were the most important factors in enhancing hospitality department students’ professional identity and innovative behavior.

## Introduction

The COVID-19 pandemic severely impacted employment in the tourism and hospitality industries, with an estimated 100–120 million people worldwide becoming unemployed at the start of the pandemic (The World Tourism Organization [UNWTO], 2021). According to the “State of the Hotel Industry 2021” report, issued by the American Hotel & Lodging Association [AHLA] (2021), 2020 was a devastating year for the hospitality industry, with historically low occupancy rates, massive job losses, and hotel closures across the United States. Hotels were among the foremost industries heavily impacted by the pandemic and would be among the last to recover. All signs indicate that the hotel industry would move toward recovery in 2022, but a full recovery would take several years. The severe impact on the hospitality and tourism industries spilled over into hospitality and tourism education (Joshi and Gupta, 2021).

A core trend in the hospitality industry is the application and development of innovations that provide a strong impetus for this industry’s development (Dzhandzhugazova et al., 2016). Diversified innovative behavior contributes to the trend of innovation in the hospitality industry, ensuring the successful development of hotels (Zaitseva, 2013). Therefore, innovation is crucial in the pursuit for sustainable development (Kearney et al., 2009). Moreover, students’ innovative behaviors play a key role in the competitive environment of the hotel industry (Dzhandzhugazova et al., 2016).

The conceptual development of professional identity is an inherent aspect of professionalism (Dubar, 1991). The development of one’s professional identity requires the higher-order thinking process of generating self-awareness (Garrison et al., 2001). Marhuenda et al. (2004) believed that socialization is a key element in the improvement of tourism professional identity, which provides a sense of belonging to the group. Students’ professional identity is constituted by attitudes, values, knowledge, and beliefs about the profession (Adams et al., 2006), which are important factors that motivate students to continue in the profession. Fingerhut et al. (2021) stated that aesthetic taste is related to personal identity. While professional identity can be theorized in terms of emotions, attitudes, and values (Sabanciogullari and Dogan, 2015), aesthetic experience also represents personal attitudes, emotions, feelings, and values (Schellekens, 2009; Saito, 2017; Korpelainen, 2021). Therefore, the acquisition of aesthetic experience enhances the professional identity of personal attitudes and values. Aesthetic experience can contribute to the generation of innovative behaviors in students (Chang and Jaisook, 2021), and it is an important concept for sustainable development (Korpelainen, 2021).

Hospitality and tourism department students’ innovative behaviors are nurtured in school (Chang, 2018), which is an environment that fosters students’ aesthetic experience (Chang and Jaisook, 2021; D’Olimpio, 2021). Over time, a supportive learning environment can promote students’ personal and professional identities. According to social identity theory (SIT), the social environment can change an individual’s behavior as long as the individual is able to modify their self-identity or self-concept that derives from the knowledge of and emotional attachment to social groups (Tajfel, 1971). Therefore, school climate may stimulate students’ professional identity (Bizumic et al., 2009; Reynolds et al., 2017), aesthetic experience (Caiman and Jakobson, 2021), and innovative behavior (Kleebbua and Lindratanasirikul, 2021). Moreover, the interaction between school climate and students’ aesthetic experience may also affect students’ professional identity and innovative behavior.

However, as students are nested within classes or schools, appropriate analytical methods such as multilevel modeling and hierarchical linear modeling (HLM) must be used; otherwise, the data will be confounded by hierarchical relationships and lead to misleading findings (Raudenbush and Bryk, 2002). Therefore, considering that students’ aesthetic experience, professional identity, and innovative behavior are nested within school climate, HLM is used in this study. Little research has been conducted on hospitality department students’ professional identity and aesthetic experience in higher education institutions (HEIs), but this is important for their future engagement in the hospitality industry and their sustainable development. China’s HEIs include public degree-granting universities, vocational colleges, and junior colleges. Therefore, we investigated the influences of school climate and students’ aesthetic experience on professional identity and innovative behavior in the hospitality department of the HEIs in Hainan, China. This study will help students to join the hospitality industry after graduation and develop a long-term career in the industry.

## Literature review

### Professional identity and aesthetic experience

Professional identity can be theorized in terms of emotions, attitudes, and values (Sabanciogullari and Dogan, 2015). The development of a professional identity is an inherent aspect of professionalism, a self-awareness stemming from reflective practice (Dubar, 1991). According to the connotation of SIT proposed by Tajfel (1971), individual self-concept is composed of personal and social identities. The former refers to individual characteristics or personality, which is different for each person, and the latter refers to an individual’s perception of belonging to a certain social group. Social identity processes can also unite individuals through psychological associations with similar or shared social categories (James, 2015). Moreover, social processes are important for identity formation (Blue et al., 2011; Cruess et al., 2015). Marhuenda et al. (2004) pointed out that social processes are essential also for tourism professionals’ identity formation because a professional identity provides a sense of belonging to a group.

According to Wiles (2013), professional identity is not just about observing and demonstrating certain traits, competences, and values but also about the process by which students combine their own experiences and identify themselves as professionals. Therefore, personal experience, reflection on experience, and knowledge about one’s discipline are powerful sources of professional identity (Henkel, 2005; Steinart et al., 2007). Whereas aesthetic experience is related to personal identity (Fingerhut et al., 2021), it also includes personal attitudes, feelings, and values (Saito, 2017; Korpelainen, 2021). Dangmei (2017) stated that aesthetics refer to sensory knowledge and the felt meaning of objects and experiences, and it includes information and meaning derived based on one’s sensory experiences about feelings and emotions (Hansen et al., 2007). Aesthetic experience is also the conscious and subconscious experience that individuals have when they appreciate things of beauty (Maquet, 1986). To be a part of an aesthetic experience, beauty must transcend from its extrinsic to intrinsic values (Marković, 2012). In other words, ugly things can also elicit aesthetic experience (Eco, 2007), and good can be found in bad things (Chang and Jaisook, 2021). Thus, aesthetic experience is not only about viewing extrinsic beauty but also includes personal reflections, attitudes, and moral values (Garrison, 1997; Eaton, 2001). Aesthetic experience also encompasses emotion. Applied in education, teaching and learning signifies the importance of enlarging students’ connections to self and others to become a part of “an expansive circle of relationships between ourselves and others” (Garrison, 1997, p. 38). The emotions elicited in aesthetics come not only from simple perceptions but also from the assessment of experiences through intra-community utterances (Maitlis et al., 2013). Yu and Wang (2018) argued that aesthetic experiences are unique means of knowing oneself and the world that can stimulate the discovery and formation of identity. Moreover, one’s self-identity can be reflected and developed through powerful aesthetic experiences (Ferrucci, 2010; Yu and Wang, 2018).

### School climate and professional identity

The school climate includes social processes such as teacher support, student support, and opportunities for autonomy in the classroom (Jia et al., 2009). Teacher support refers to the emotional, academic, and social support provided by teachers (Colarossi and Eccles, 2003). Student support is the perceived emotional support, trust, and concern between students (Loukas et al., 2006). Opportunities for autonomy in the classroom are opportunities for students to make choices and decisions in learning and classroom activities. Loukas (2007) posited that the feelings and attitudes that are elicited by school environment are referred to as school climate, which encompasses physical, social, and academic measures. In the social identity approach, students’ professional identity is considered a psychological mechanism that is shaped by the school climate and learning experience. School climate includes norms, values, and expectations (Haynes et al., 1997; Petrie, 2014) that affect students’ learning, social adjustment (Brand et al., 2008), and mental health outcomes (Brand et al., 2003). Moreover, schools as holistic centers that usually emphasize academic priorities, support student–teacher relationships, and share values and approaches. Thus, school climate facilitates students’ professional identity (Bizumic et al., 2009; Maxwell et al., 2017; Reynolds et al., 2017) and contributes to students’ aesthetic experience (Caiman and Jakobson, 2021).

### School climate, aesthetic experience, and innovative behavior

Schools provide the foundation for stimulating and nurturing students’ aptitude for knowledge and innovation, and help them meet future employment challenges (Chang, 2018). Individual innovative behavior, however, refers to the individual behavior of identifying problems, generating innovative ideas or solutions, seeking support for innovative ideas, putting them into practice, and finally forming commercial products or services (Scott and Bruce, 1994). Kleysen and Street (2001) considered individual innovative behavior the implementation of ideas. Shalley et al. (2004) also argued that innovative behavior includes both the generation and implementation of new ideas. However, innovative behavior is related to unique factors such as motivation and organizational climate (Tierney and Farmer, 2011). Teachers are role models of creativity in the classroom and students learn from their teachers’ creative personalities and behaviors (Cropley, 1994). A supportive and autonomous environment creates and maintains an atmosphere of mutual respect, inclusion, openness to criticism, and innovation (Gorshunova et al., 2014). Kleebbua and Lindratanasirikul (2021) also found that the learning climate has a significant direct effect on students’ innovative behavior (Chang and Yang, 2012).

Lussier (2010) stated that the acquisition of aesthetic experience by students is positive for the development of innovative behavior (Chang and Jaisook, 2021). After an individual’s digestion, accumulation, and internalization of aesthetic experience, it may boost one’s confidence to accept challenges and becomes an essential factor for the emergence of creative behaviors (Davies et al., 2009). Aesthetic experience involves a complex interplay of the interaction of cognitive and emotional processes (Leder et al., 2004), and it consists of multiple processes that occur through interaction (Xenakis and Arnellos, 2014). Viewers absorb and internalize aesthetics through the process of contemplation and transform it into personal thinking and feedback experience, which becomes aesthetic experience and one’s personal perception of beauty (Lussier, 2010; Chang and Jaisook, 2021). This can stimulate intrinsic motivation and self-confidence, enhance one’s everyday imagination and creativity, and boost innovative behavior (Davies et al., 2009; Lussier, 2010). Aesthetic experience makes human reasoning possible, thus creating a new sense of reality. Emotions and imagination are integrated in the intellect at the time of an aesthetic experience (Garrison, 1997). Moreover, Webster and Wolfe (2013) also found that aesthetic experiences in the classroom affect students’ ability to think, explore, create, and innovate. Researchers have studied individuals’ creativity and innovative behavior from the perspective of the environment and found that the interaction between environment and individual cognition can impact individual creativity and innovative behavior (Hunter et al., 2007; Bammens, 2016). Learning contexts and aesthetics can stimulate curiosity, creativity, and innovation (Gurnon et al., 2013). In summary, school climate and aesthetic experiences affect students’ professional identity and innovative behavior; students’ aesthetic experiences may have a mediating effect between school climate with students’ professional identity and innovative behavior; and school climate may also have a moderating effect. Therefore, this study investigates the relationships between school climate, students’ aesthetic experiences, and professional identity and innovative behavior among the students from hospitality-related departments of colleges and universities.

## Materials and methods

### Research framework

**FIGURE 1:**
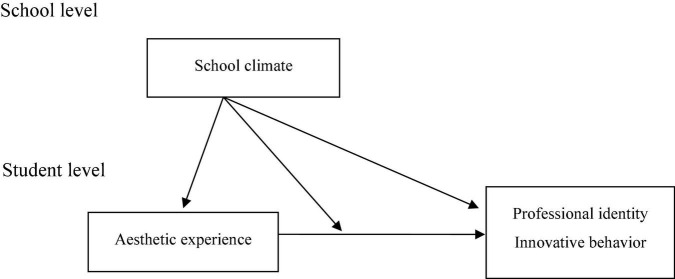
Research framework.

This study aimed to investigate the effects of school climate and students’ aesthetic experiences on professional identity and innovative behavior. We used SIT as the theoretical basis and HLM to conduct analysis. After the literature review, we proposed the research framework as follows (Figure 1).

### Research subject and sampling method

The tourism industry in Hainan, China, has been developing rapidly in recent years—particularly the hospitality industry, which requires a large number of human resources, and hospitality department students are the main force of this industry. It is important that students be able to enter the hospitality industry smoothly and sustain their careers in the industry.

This study selected students from hospitality-related departments of colleges and universities in Hainan, China, as the sample. The survey was conducted with the consent of teachers, who were asked to explain the purpose and procedure of the study to students willing to take the survey; further, teachers informed the participants that the survey was anonymous and voluntary. The survey was conducted in 25 classes of hospitality-related departments in 15 colleges and universities, with 15–20 students per class. A total of 426 students were surveyed. After removing the invalid questionnaires, there were 25 classes with 385 valid questionnaires.

### Research tools

#### School climate

The school climate scale proposed by Jia et al. (2009) was used. It comprises three dimensions: teacher support, student support, and opportunities for autonomy in the classroom. In the questionnaire, the reverse-worded items of the student support dimension were removed. A total of 18 questions were scored on a 5-point Likert scale. According to reliability analysis, the total Cronbach’s α = 0.956. The formal questionnaire was subjected to confirmatory factor analysis (CFA), in which the factor loadings of teacher support ranged from 0.730 to 0.837, with construct reliability (CR) = 0.923 and average variance extracted (AVE) = 0.630; the factor loadings of opportunities for autonomy ranged from 0.729 to 0.915, with CR = 0.925 and AVE = 0.711; and the factor loadings of student support ranged from 0.820 to 0.897, with CR = 0.947 and AVE = 0.749. The factor loadings of all the items were greater than 0.45, indicating that convergent validity had been achieved (Bentler and Wu, 1995). The CR values of all the items exceeded the evaluation criteria of 0.70, and the AVE values of all the items exceeded 0.50, indicating good composite reliability and construct validity (Fornell and Larcker, 1981). As for the overall goodness of fit (GOF) of the scale, standardized root mean square residual (SRMR) = 0.0612, χ^2^/*df* = 5.057, root mean square error of approximation (RMSEA) = 0.103, goodness fit index (GFI) = 0.837, adjusted goodness of fit index (AGFI) = 0.789, parsimony goodness of fit index (PGFI) = 0.646, normed fit index (NFI) = 0.896, incremental fit index (IFI) = 0.915, comparative fit index (CFI) = 0.915, parsimony normed fit index (PNFI) = 0.773, and parsimony comparative fit index (PCFI) = 0.789; thus, most of these values meet the criteria (Hair, 1998), indicating that the scale has adequate GOF.

#### Aesthetic experience

The students’ aesthetic experience scale proposed by Chang (2017) was used in this study. It includes the following themes: “pleasure of beauty” and “aesthetic attitude,” “understanding of beauty,” and “full experience”; it comprises 21 questions and uses a 5-point Likert scale. The reliability analysis revealed that the Cronbach’s α = 0.976. According to the CFA results, the factor loadings of pleasure of beauty ranged from 0.817 to 0.914, with CR = 0.947 and AVE = 0.750; the factor loadings of aesthetic attitude ranged from 0.836 to 0.931, with CR = 0.947 and AVE = 0.781; the factor loadings of understanding of beauty ranged from 0.847 to 0.876, with CR = 0.934 and AVE = 0.740; and the factor loadings of full experience ranged from 0.830 to 0.901, with CR = 0.932 and AVE = 0.733. As for the overall GOF of the scale, SRMR = 0.0412, χ^2^/*df* = 4.774, RMSEA = 0.099, GFI = 0.817, AGFI = 0.769, PGFI = 0.647, NFI = 0.907, IFI = 0.925, CFI = 0.925, PNFI = 0.790, and PCFI = 0.806; thus, most of the values meet the criteria.

#### Professional identity

The students’ professional identity scale proposed by Yu et al. (2021) was used. It has three dimensions, namely, “professional cognition,” “professional appraisal,” and “professional emotion”; the scale comprises 14 questions and uses a 5-point Likert scale. The reliability analysis showed that the total Cronbach’s α = 0.951. According to the results of CFA, the factor loadings of professional cognition ranged from 0.803 to 0.894, with CR = 0.917 and AVE = 0.735; the factor loadings of professional appraisal ranged from 0.743 to 0.901, with CR = 0.896 and AVE = 0.685; the factor loadings of professional emotion ranged from 0.730 to 0.893, with CR = 0.934 and AVE = 0.705. As for the overall GOF of the scale, SRMR = 0.0524, χ^2^/*df* = 5.922, RMSEA = 0.113, GFI = 0.865, AGFI = 0.809, PGFI = 0.610, NFI = 0.910, IFI = 0.924, CFI = 0.924, PNFI = 0.740, and PCFI = 0.752.

#### Innovative behavior

The students’ innovative behavior scale proposed by Chang (2018) was used with a total of 12 questions scored using a 5-point Likert scale. The reliability analysis showed that the total Cronbach’s α = 0.955. According to the results of CFA, the factor loadings ranged from 0.661 to 0.876, with CR = 0.955 and AVE = 0.639. For the overall GOF of the scale, SRMR = 0.0592, χ^2^/*df* = 9.432, RMSEA = 0.148, GFI = 0.826, AGFI = 0.718, PGFI = 0.509, NFI = 0.897, IFI = 0.907, CFI = 0.907, PNFI = 0.652, and PCFI = 0.659.

## Results

This study investigated the relationships between aesthetic experience and professional identity in school climate and students’ innovative behavior. Therefore, a cross-level research was conducted by using escalation of the unit of analysis (Kark et al., 2003), wherein students filled out the school climate scale and then elevated the completed school climate scale to the school level for analysis, which could reduce common method variance (Craighead et al., 2011).

### Intra-class correlation coefficient statistics

In the study, the outcome variables were professional identity and innovative behavior, which were subjected to a null model test to calculate the intra-class correlation coefficient (ICC) so as to confirm the need for multilevel analysis (Raudenbush and Bryk, 2002). The analysis results, the ICC values of professional cognition, professional appraisal, professional emotion, and innovative behavior were 0.101, 0.097, 0.141, and 0.067, respectively, with Chi-square (χ^2^) critical values ranging from 50.603 to 87.22, all reaching the significance level of 0.05. Thus, it was suitable for multilevel analysis. Before conducting HLM analysis, it was necessary to detect the presence of intergroup variation (Girod and Wong, 2002) in the data before individual-level data could be aggregated to the group level. The mean value r*_*wgj*_* of school climate was calculated to be 0.16, justifying this aggregation procedure.

### Random parameter regression model

To understand whether students’ aesthetic experience has a direct effect on professional identity and innovative behavior respectively, we conducted analysis using the following model. To keep the paper reasonably concise, we took only professional cognition as an example to elaborate. The effects of the rest of the dimensions on innovative behavior were analyzed in the same way.

Level-1: professional cognition_*ij*_ = β_0*j*_ + β_1*j*_ × (pleasure of beauty_*ij*_) + β_2*j*_ × (aesthetic attitude_*ij*_) + β_3*j*_ × (understanding of beauty_*ij*_) + β_4*j*_ × (full experience_*ij*_) + r_*ij*_

Level-2: β_0*j*_ = γ_00_ + u_0*j*_


$$\begin{array}{l} \beta_{1j}=\gamma_{10}+u_{1j}\\ \beta_{2j}=\gamma_{20}+u_{2j}\\ \beta_{3j}=\gamma_{30}+u_{3j}\\ \beta_{4j}=\gamma_{40}+u_{4j} \end{array}$$

According to the analysis results (as shown in Table 1), in the professional cognition section, full experience in aesthetic experience reached a significant level (γ_40_ = 0.501, *p* = 0.000). In the professional appraisal section, understanding of beauty and full experience reached a significant level (γ_30_ = 0.349, *p* = 0.008, γ_40_ = 0.395, *p* = 0.018); in the professional emotion section, full experience reached a significant level (γ_40_ = 0.387, *p* = 0.001). In the innovative behavior section, understanding of beauty and full experience reached a significant level (γ_30_ = 0.375, *p* = 0.000; γ_40_ = 0.271, *p* = 0.02). As for the explained variation (*R*^2^) for aesthetic experience, at the individual level, it was calculated that in level-1, the *R*^2^ values of professional cognition, professional appraisal, professional emotion, and innovative behavior were 41.39, 36.54, 38.74, and 52.28%, respectively.

**TABLE 1:** Summary of random parameter regression model.

Fixed effect	Professional cognition		Professional appraisal		Professional emotion		Innovative behavior	
				
	γ	SE	γ	SE	γ	SE	γ	SE
Pleasure of beauty γ_10_	–0.072	0.13	–0.270	0.12	0.275	0.14	–0.027	0.09
Aesthetic attitude γ_20_	0.060	0.09	0.122	0.11	–0.051	0.19	0.047	0.11
Understanding of beauty γ_30_	0.227	0.12	0.349**	0.12	0.019	0.09	0.375***	0.09
Full experience γ_40_	0.501***	0.10	0.395**	0.15	0.387**	0.098	0.271*	0.11

**Random effect**	**Variance**	***p***	**Variance**	***p***	**Variance**	***p***	**Variance**	***p***

γ_ij_	0.285		0.323		0.274		0.164	
U_1j_	0.232	>0.500	0.086	>0.500	0.309	0.034	0.066	0.108
U_2j_	0.078	>0.500	0.060	>0.500	0.552	0.005	0.101	0.016
U_3j_	0.177	0.155	0.128	0.221	0.033	0.400	0.084	0.228
U_4j_	0.063	>0.500	0.302	0.008	0.047	>0.500	0.130	0.232
U_0j_	0.069	0.000	0.067	0.000	0.084	0.000	0.037	0.000

*p < 0.05, **p < 0.01, ***p < 0.001.

### Direct effect of school climate

To further verify whether the presence of the intercept term could be explained by the level-2 variable (i.e., school climate), we conducted analysis using the following model.

Level-1: professional cognition_*ij*_ = β_0j_ + β_1j_ × (pleasure of beauty_*ij*_) + β_2j_ × (aesthetic attitude_*ij*_) + β_3*j*_ × (understanding of beauty_*ij*_) + β_4*j*_ × (full experience_*ij*_) + r_*ij*_

Level-2: β_0*j*_ = γ_00_ + γ_01_ × (teacher support_*j*_) + γ_02_ × (opportunities for autonomy_*j*_) + γ_03_ × (student support_*j*_) + u_0*j*_


$$\begin{array}{l} \beta_{1j}=\gamma_{10}+u_{1j}\\ \beta_{2j}=\gamma_{20}+u_{2j}\\ \beta_{3j}=\gamma_{30}+u_{3j}\\ \beta_{4j}=\gamma_{40}+u_{4j} \end{array}$$

According to the analysis results (as shown in Table 2), in the professional cognition section, teacher support in school climate reached the significant level (γ_01_ = 0.758, *p* = 0.000); and in the professional appraisal section, teacher support reached the significant level (γ_01_ = 1.023, *p* = 0.001); and in the professional emotion section, teacher support reached the significant level (γ_01_ = 1.024, *p* = 0.002). In the innovative behavior section, teacher support, opportunities for autonomy, and student support all reached the significant level (γ_01_ = 0.335, *p* = 0.014; γ_02_ = 0.365, *p* = 0.012; γ_03_ = 0.240, *p* = 0.036). From the above, it is clear that in all cases, teacher support has a direct effect on professional identity, especially innovative behavior.

**TABLE 2:** Summary of intercept prediction model.

Fixed effect	Professional cognition		Professional appraisal		Professional emotion		Innovative behavior	
				
	γ	SE	γ	SE	γ	SE	γ	SE
Pleasure of beauty γ_10_	–0.143	0.14	−0.255*	0.12	0.252	0.14	–0.066	0.09
Aesthetic attitude γ_20_	0.100	0.09	0.094	0.12	–0.056	0.17	0.041	0.11
Understanding of beauty γ_30_	0.260*	0.11	0.396*	0.11	0.051	0.09	0.420***	0.08
Full experience γ_40_	0.485***	0.09	0.348*	0.14	0.365**	0.10	0.261*	0.11
Teacher support γ_01_	0.758***	0.13	1.023**	0.27	1.024**	0.29	0.335*	0.13
Opportunities for autonomy γ_02_	0.246	0.15	–0.240	0.28	0.206	0.24	0.365*	0.13
Student support γ_03_	0.176	0.12	0.024	0.19	–0.045	0.15	0.240*	0.11

**Random effect**	**Variance**	***p***	**Variance**	***p***	**Variance**	***p***	**Variance**	***p***

γ_ij_	0.275		0.319		0.269		0.161	
U_1j_	0.288	>0.500	0.104	>0.500	0.289	0.035	0.071	0.103
U_2j_	0.067	>0.500	0.060	>0.500	0.527	0.004	0.117	0.013
U_3j_	0.154	0.129	0.111	0.228	0.063	0.403	0.063	0.252
U_4j_	0.055	>0.500	0.280	0.005	0.045	>0.500	0.166	0.209
U_0j_	0.015	0.053	0.036	0.000	0.019	0.003	0.004	0.273

*p < 0.05, **p < 0.01, ***p < 0.001.

### Multilevel mediating effect

The first step to test the multilevel mediating effects was to examine whether professional identity, innovative behavior, and aesthetic experience could be effectively explained by the overall level of school climate. In Equation 1, it was considered important that the estimated value of γ*^c^*_01_ has a significant level; if γ*^c^*_01_ is significant, it indicates that there is a mediating effect of school climate on pleasure of beauty in aesthetic experience. In Equation 2, it was considered important that the estimated value of γ*^a^*_01_ has a significant level; only when it reached a significant level could we proceed with the test. We listed only Equations 1, 2 for pleasure of beauty and professional cognition.

Professional cognition_*ij*_ = β*^c^*_0*j*_+r*^c^*_*ij*_


(1) $$\begin{array}{l} \beta_{0\text{j}}^{\text{c}}=\gamma_{00}^{\text{c}}+\gamma_{01}^{\text{c}}{\text{ teacher support}}_{\text{j}}+\gamma_{02}^{\text{c}}\text{ opportunities for}\\ \;\;\;\;\;{\text{autonomy}}_{\text{j}}+\gamma_{03}^{\text{c}}{\text{ student support}}_{\text{j}}+{\text{U}}_{0\text{j}}^{\text{c}} \end{array}$$

Pleasure of beauty_*ij*_ = β*^a^*_0*j*_+r*^a^*_*ij*_


(2) $$\begin{array}{l} \beta_{0\text{j}}^{\text{a}}=\gamma_{00}^{\text{a}}+\gamma_{01}^{\text{a}}{\text{ teacher support}}_{\text{j}}+\gamma_{02}^{\text{a}}\text{ opportunities for}\\ \;\;\;\;\;{\text{autonomy}}_{\text{j}}+\gamma_{03}^{\text{a}}{\text{ student support}}_{\text{j}}+{\text{U}}_{0\text{j}}^{\text{a}} \end{array}$$

According to the analysis results (as shown in Table 3), γ_01_ of professional cognition, professional appraisal, and professional emotion reached a significant level (γ*^c^*_01_ = 0.869, SE = 0.20, *p* = 0.000; γ*^c^*_01_ = 0.964, SE = 0.30, *p* = 0.005; γ*^c^*_01_ = 1.076, SE = 0.36, *p* = 0.007); further, γ_01_ and γ_03_ of innovative behavior reached a significant level (γ*^c^*_01_ = 0.324, SE = 0.15, *p* = 0.040; γ*^c^*_03_ = 0.339, SE = 13, *p* = 0.014). As shown in Table 4, in the pleasure of beauty section, γ_01_ and γ_03_ reached a significant level (γ*^a^*_01_ = 0.486, SE = 0.20, *p* = 0.027; γ*^a^*_03_ = 0.645, SE = 14, *p* = 0.000); in the aesthetic attitude section, γ_01_ and γ_03_ reached a significant level (γ*^a^*_01_ = 0.350, SE = 17, *p* = 0.021; γ*^a^*_03_ = 0.752, SE = 10, *p* = 0.000); in the understanding of beauty section, γ_01_ and γ_03_ reached a significant level (γ*^a^*_01_ = 0.340, SE = 0.14, *p* = 0.025; γ*^a^*_03_ = 0.672, SE = 12, *p* = 0.000); and finally, in the full experience section, γ_03_ reached a significant level (γ*^a^*_03_ = 0.959, SE = 13, *p* = 0.000).

**TABLE 3:** Summary of hierarchical linear modeling analysis of learning environment with professional identity and innovative behavior.

Fixed effect	Professional cognition		Professional appraisal		Professional emotion		Innovative behavior	
				
	γ	SE	γ	SE	γ	SE	γ	SE
Teacher support γ*^c^*_01_	0.869***	0.20	0.964**	0.30	1.076**	0.36	0.324*	0.15
Opportunities for autonomy γ*^c^*_02_	0.243	0.24	–0.40	0.35	0.146	0.28	0.286	0.16
Student support γ*^c^*_03_	0.010	0.19	–0.160	0.20	–0.047	0.20	0.339*	0.13

**Random effect**	**Variance**	***p***	**Variance**	***p***	**Variance**	***p***	**Variance**	***p***

γ_ij_	0.482		0.509		0.446		0.335	
U_0j_	0.000	>0.500	0.023	0.025	0.009	0.203	0.000	>0.500

*p < 0.05, **p < 0.01, ***p < 0.001.

**TABLE 4:** Summary of hierarchical linear modeling analysis of learning environment and aesthetic experience.

Fixed effect	Pleasure of beauty		Aesthetic attitude		Understanding of beauty		Full experience	
				
	γ coefficient	SE	γ coefficient	SE	γ coefficient	SE	γ coefficient	SE
Teacher support γ^a^_01_	0.486*	0.20	0.350*	0.17	0.340*	0.14	0.207	0.18
Opportunities for autonomy γ^a^_02_	–0.238	0.18	–0.041	0.18	0.064	0.17	–0.318	0.19
Student support γ^a^_03_	0.645***	0.14	0.752***	0.01	0.672***	0.12	0.959***	0.13

**Random effect**	**Variance**	***p***	**Variance**	***p***	**Variance**	***p***	**Variance**	***p***

γ_ij_	0.410		0.413		0.386		0.368	
U_0j_	0.000	>0.500	0.000	>0.500	0.000	>0.500	0.000	>0.500

*p < 0.05, ***p < 0.001.

The second step was to further put both the higher-level explanatory variables and the mediating variables into the equation to test the explanatory power of school climate and aesthetic experience on professional identity and innovative behavior. Our focus was on the significance test of γ*^c^*′_01_ (Z→Y) in Equation 3a. The model was as follows:

Professional cognition_*ij*_ = β*^b^*_0*j*_ + β*^b^*_1*j*_ × (pleasure of beauty_*ij*_) + β*^b^*_2*j*_ × (aesthetic attitude_*ij*_) + β*^b^*_3*j*_ × (understanding of beauty_*ij*_) + β*^b^*_4*j*_ × (full experience_*ij*_) + r*^b^*_*ij*_


(3) $$\begin{array}{l} \beta_{0\text{j}}^{\text{b}}=\gamma_{00}^{\text{b}}+\gamma_{01}^{\text{c'}}\times \left({\text{teacher support}}_{\text{j}}\right)+\gamma_{02}^{\text{c'}}\\ \;\;\;\;\;\times \left({\text{opportunities for autonomy}}_{\text{j}}\right)+\gamma_{03}^{\text{c'}}\\ \;\;\;\;\;\times \left({\text{student support}}_{\text{j}}\right)+{\text{U}}_{0\text{j}}^{\text{b}} \end{array}$$

(3a) $$\begin{array}{l} \beta_{1\text{j}}^{\text{b}}=\gamma_{10}^{\text{b}}\\ \beta_{\text{2j}}^{\text{b}}=\gamma_{20}^{\text{b}}\\ \beta_{\text{3j}}^{\text{b}}=\gamma_{30}^{\text{b}}\\ \beta_{\text{4j}}^{\text{b}}=\gamma_{40}^{\text{b}} \end{array}$$

After adding mediating variables, the results were obtained from the intercept prediction model, as shown in in Table 2. In terms of professional cognition, γ*^c^*′_01_ of teacher support decreased to 0.758 and also reached a significant level (*t* = 5.827, *p* = 0.000), indicating a partial mediating effect. Among the mediated variables, only understanding of beauty and full experience reached a significant level (γ*^b^*_30_ = 0.260, *t* = 2.281, *p* = 0.032; γ*^b^*_40_ = 0.485, *t* = 5.21, *p* = 0.000). Thus, understanding of beauty and full experience had partial mediating effects between teacher support and professional cognition. In the professional appraisal section, γ*^c^*′_01_ of teacher support increased to 1.023 and also reached a significant level (*t* = 3.764, *p* = 0.001); thus, it has no mediating effect. For the professional emotion section, γ*^c^*′_01_ of teacher support decreased to 1.024 and reached a significant level (*t* = 3.548, *p* = 0.002); thus, it has a partially mediating effect. Among the mediating variables, only full experience reached a significant level (γ*^b^*_40_ = 0.365, *t* = 3.80, *p* = 0.001). Thus, full experience has a partial mediating effect. In the innovative behavior section, γ*^c^*′_01_ of teacher support increased to 0.335 and also reached a significant level (*t* = 2.683, *p* = 0.014); thus, it has no mediating effect. γ*^c^*′_03_ of student support decreased to 0.24 and reached a significant level (*t* = 3.548, *p* = 0.002); thus, it has a partial mediating effect. Among the mediating variables, understanding of beauty and full experience reached a significant level (γ*^b^*_30_ = 0.420, *t* = 5.051, *p* = 0.000; γ*^b^*_40_ = 0.261, *t* = 2.328, *p* = 0.029); thus understanding of beauty and full experience have partial mediating effects.

### Moderating effect of school climate

To verify whether the presence of the slope term could be explained by the level-2 variable (i.e., school climate), we conducted analysis using the following model.

Level-1: professional cognition_ij_ = β_0j_ + β_1j_ × (pleasure of beauty_ij_) + β_2j_ × (aesthetic attitude_ij_) + β_3j_ × (understanding of beauty_ij_) + β_4j_ × (full experience_ij_) + r_ij_

Level-2: β_0j_ = γ_00_ + γ_01_ × (teacher support_j_) + γ_02_ × (opportunities for autonomy_j_) + γ_03_ × (student support_j_) + u_0j_

β_1j_ = γ_10_ + γ_11_ × (teacher support_j_) + γ_12_ × (opportunities for autonomy_j_) + γ_13_ × (student support_j_) + u_1j_

β_2j_ = γ_20_ + γ_21_ × (teacher support_j_) + γ_22_ × (opportunities for autonomy_j_) + γ_23_ × (student support_j_) + u_2j_

β_3j_ = γ_30_ + γ_31_ × (teacher support_j_) + γ_32_ × (opportunities for autonomy_j_) + γ_33_ × (student support_j_) + u_3j_

β_4j_ = γ_40_ + γ_41_ × (teacher support_j_) + γ_42_ × (opportunities for autonomy_j_) + γ_43_ × (student support_j_) + u_4j_

As shown in Table 5, in the professional cognition section, the interaction coefficient between aesthetic attitude and teacher support reached a significant level (γ_21_ = −1.457, SE = 0.55, *p* = 0.016). This indicates that teacher support in school climate has a negative moderating effect on the relationship between aesthetic attitude and professional cognition at the individual level. The interaction coefficient between full experience and student support was significant (γ_43_ = 0.908, SE = 0.33, *p* = 0.013). This indicates that student support has a positive moderating effect on the relationship between full experience and professional cognition. In the professional appraisal section, the interaction coefficient between full experience and student support was significant (γ_43_ = 1.705, SE = 0.68, *p* = 0.021). This indicates that student support has a positive moderating effect on the relationship between full experience and professional appraisal. In the professional emotion section, the interaction between full experience and teacher support is significant (γ_41_ = 2.092, SE = 0.52, *p* = 0.001). This indicates that teacher support positively moderates the relationship between full experience and professional emotion; that is, teacher support reinforces the relationship between full experience and professional emotion. The interaction coefficient between full experience and opportunities for autonomy is significant (γ_42_ = −1.930, SE = 0.50, *p* = 0.001). This indicates that the variable opportunities for autonomy has a negative moderating effect between full experience and professional emotion; that is, opportunities for autonomy at the organizational level would weaken the relationship between full experience and professional emotion. In the innovative behavior section, there was no interaction of school climate with aesthetic experience and innovative behavior.

**TABLE 5:** Summary of slope prediction model.

Fixed effect	PC		PA		PE		IB	
				
	γ	SE	Γ	SE	γ	SE	γ	SE
PBγ_10_	–1.006	2.16	0.1556	2.32	–2.933	3.18	–3.219	1.49
AAγ_20_	2.816	1.83	1.339	2.57	3.394	3.67	3.28	2.02
UBγ_30_	3.494	2.02	2.546	2.12	2.547	1.60	2.06	1.41
FEγ_40_	−4.183*	1.04	–4.396	2.65	−2.657*	1.08	–1.847	2.10
TSγ_01_	0.878*	0.19	0.948*	0.30	1.045*	0.34	0.320*	0.15
OAγ_02_	0.255	0.24	–0.022	0.35	0.173	0.27	0.285	0.16
SSγ_03_	–0.022	0.18	–0.183	0.21	–0.053	0.19	0.344*	0.13
PB × TSγ_11_	0.982	0.80	0.035	0.85	1.147	1.25	0.866	0.68
PB × OAγ_12_	0.330	1.05	1.219	0.82	–0.991	1.24	–0.341	0.81
PB × SSγ_13_	–1.026	0.85	–1.272	0.84	0.650	1.00	0.305	0.78
AA × TSγ_21_	−1.457*	0.55	–0.773	1.01	–2.306	1.56	–1.535	0.92
AA × OAγ_22_	1.018	0.60	1.049	0.99	2.907	1.69	1.76	0.87
AA × SSγ_23_	–0.243	0.42	–0.543	0.80	–1.362	1.16	–0.992	0.75
UB × TSγ_31_	0.921	0.82	0.136	0.77	–0.802	0.60	0.077	0.60
UB × OAγ_32_	–1.515	0.75	–0.545	0.86	0.224	0.56	–0.768	0.48
UB × SSγ_33_	–0.304	0.46	0.079	0.55	–0.080	0.51	0.210	0.52
FE × TSγ_41_	–0.102	0.44	1.018	0.85	2.092**	0.52	0.290	0.89
FE × OAγ_42_	0.411	0.38	1.576	0.95	−1.930**	0.50	–0.696	0.79
FE × SSγ_43_	0.908*	0.33	1.705*	0.68	0.540	0.45	0.911	0.63

**Random effect**	**Variance**	***p***	**Variance**	***p***	**Variance**	***p***	**Variance**	***p***

γ_ij_	0.275		0.318		0.267		0.158	
U_1j_	0.257	0.223	0.078	0.353	0.380	0.018	0.106	0.068
U_2j_	0.073	>0.500	0.104	>0.500	0.646	0.004	0.158	0.017
U_3j_	0.157	0.104	0.172	0.165	0.039	>0.500	0.072	0.193
U_4j_	0.023	>0.500	0.234	0.022	0.022	>0.500	0.164	0.113
U_0j_	0.015	0.067	0.038	0.000	0.02	0.003	0.004	0.286

*p < 0.05, **p < 0.01. TS, teacher support; OA, opportunities for autonomy; SS, student support; PB, pleasure of beauty; AA, aesthetic attitude; UB, understanding of beauty; FE, full experience; PC, professional cognition; PA, professional appraisal; PE, professional emotion; IB, innovative behavior.

## Discussion

### Influences of students’ aesthetic experience on professional identity and innovative behavior

The study’s results show that full experience in students’ aesthetic experience helps improve students’ professional identity, professional cognition, professional appraisal, and professional emotion. In other words, if students remember good things related to the past while engaging in creation and discuss good experiences with others, they would have a higher sense of professional identity, better understand their profession, and be more compatible with and enjoy their profession. Students can gain a stronger sense of professional identity through experience, reflection of experience, and their own disciplines (Henkel, 2005; Steinart et al., 2007). Students’ understanding of beauty can enhance their professional appraisal. It includes conceptual knowledge, skills, feelings, attitudes, behaviors, emotions, and values, where values are the values or importance that individuals perceive after comparing with other professions (Girod and Wong, 2002). Thus, students can apply their understanding of beauty in professional appraisal to have a higher and better evaluation of the hospitality profession.

Students’ understanding of beauty and full experience as part of aesthetic experience can enhance their innovation behaviors. Students can see the special and subtle aspects of beauty, express concepts and reasons for beauty, recall relevant beauty associated with creation, and share and discuss such experiences with others. Students will absorb, internalize, and transform these related aesthetics into personal thinking and experiences that can enhance intrinsic motivation and self-confidence and boost thinking, exploring the occurrence of contemplative, explorative, creative, and innovative behaviors (Davies et al., 2009; Lussier, 2010; Webster and Wolfe, 2013; Chang and Jaisook, 2021). Therefore, students with an understanding and experience of beauty would develop innovative behaviors.

### Influences of school climate on students’ professional identity and innovative behavior

Teacher support as part of school climate can enhance students’ professional identity. Teachers’ concern, trust in students, and help provided to students to solve problems make students feel a sense of belonging, thereby enhancing their professional identity (Marhuenda et al., 2004). School climate (i.e., teacher support, opportunities for autonomy, and student support) contributes to the enhancement of students’ innovative behaviors. Researchers have also pointed out the importance of a supportive environment for students’ innovation behavior (Chang, 2018; Kleebbua and Lindratanasirikul, 2021). Teacher support, student support, and opportunities for autonomy in schools enable students to have more new ideas and practices and boost their innovative behaviors. Moreover, with teachers’ support, students will be more confident in their studies and will identify more with their profession, capabilities, and values to join the hospitality-related professions in the future.

### Mediating effect of students’ aesthetic experience on the relations between school climate and professional identity on innovative behavior

Students’ understanding of beauty and full experience have partially mediating effects on teacher support and professional cognition. Teacher support can enhance students’ professional cognition through their understanding of beauty and full experience. This means that teacher support can enable students to have a deeper understanding of beauty, share beautiful experiences with others, discuss with others about careers for gaining a deeper understanding, and evaluate and increase their knowledge of the hospitality profession.

Students’ understanding of beauty and full experience partially mediates the relationship between student support and innovative behavior. Student support can lead to innovative behavior through students’ understanding of beauty and full experience. This means that mutual trust, liking, respect, and help among students at school make it easier for them to understand the styles, concepts, and special features of beauty, to discuss beauty with others, and perceive the beauty associated with creation—all of which contribute to their innovative behaviors.

### Moderating effect of school climate on the relation between students’ aesthetic experience and professional identity

Teacher support has a negative moderating effect between students’ aesthetic attitude and professional cognition. However, teacher support can enhance students’ professional cognition through students’ understanding of beauty and full experience, while students’ aesthetic attitude has no effect on professional cognition. This may be because when teacher support is stronger, the relationship between students’ aesthetic attitude and professional cognition is weakened, thus causing a negative moderating situation. This also reveals the importance of teacher support for hospitality department students’ professional cognition.

Student support has a positive moderating effect on the relationships between students’ full experience with professional cognition and professional appraisal. In other words, when students can help, trust and respect each other, as well as discuss and share good experiences with others, students’ professional cognition and professional appraisal of the hospitality industry would be higher. Teacher support has a positive moderating effect on students’ full experience and professional emotion. This indicates that teachers can help students solve problems, care about them, and believe in them, and students would recall good things about their teachers and share their good experiences with others. Thus, students evaluate the hospitality profession positively and are more willing to engage in hospitality-related work.

The variable opportunities for autonomy in school climate has a negative moderating effect on full experience and professional emotion, while full experience has a positive effect on professional emotion. The interaction between opportunities for autonomy and students’ full experience has a negative effect on students’ professional emotion. In other words, when students can decide rules in the classroom and discuss and share their good experiences with others, it decreases their satisfaction and evaluation of the hospitality profession. This may be because hospitality department students have fewer opportunities for autonomy, and opportunities for autonomy has no direct effect on full experience and professional emotion. Therefore, we should pay attention to the moderation of opportunities for autonomy in school climate.

## Conclusion

It was found that teacher support in school climate and full experience in students’ aesthetic experience are important for hospitality department students’ professional identity and can enhance students’ professional identity, which includes professional cognition, professional appraisal, and professional emotion. Students’ understanding of beauty can also enhance their professional appraisal. School climate can enhance students’ innovative behavior. Students’ understanding of beauty and full experience in aesthetic experience can also enhance students’ innovative behavior.

Furthermore, teacher support can enhance students’ professional cognition and professional emotion through students’ full experience. Teacher support can also enhance students’ professional cognition through students’ own understanding of beauty. Student support can enhance innovative behavior through students’ understanding of beauty and full experience. Therefore, understanding of beauty and full experience in students’ aesthetic experience are important mediating variables for hospitality department students’ professional identity and innovative behavior.

With regard to the moderating effect of school climate, teacher support negatively moderates the relationship between students’ aesthetic attitude and professional cognition. Student support positively moderates the relationships between students’ full experience with professional cognition and professional appraisal. Teacher support positively moderates the relationship between students’ full experience and professional emotion. Opportunities for autonomy negatively moderates the relationship between students’ full experience and professional emotion. Therefore, teacher support and student support in school climate have an important moderating effect on hospitality department students’ professional identity.

In conclusion, for hospitality students, teacher support and student support in school climate and understanding of beauty and full experience in students’ aesthetic experience are crucial for enhancing students’ professional identities and innovative behaviors. Hospitality-related departments in colleges and universities should prioritize teacher support and student support in addition to students’ understanding of beauty and full experience to enhance students’ professional identity and innovative behavior. This will aid students to enter the hospitality industry swiftly after graduation and sustain their careers in the industry. Therefore, it is recommended that hospitality-related departments in colleges and universities should prioritize teacher support and student support and incorporate aesthetic-related courses to give students an understanding and full experience of beauty. The purpose is to improve students’ identification with the hospitality profession, including professional cognition, professional appraisal, and professional emotion, and enhance their innovative behaviors. Accordingly, they can enter the hospitality industry swiftly after graduation and attain sustainable career development in the industry.

With respect to research recommendations, this study investigates the effect of school climate on the relation between hospitality students’ professional identities and innovative behaviors among students at hospitality-related departments in colleges and universities during the COVID-19 pandemic in 2022, without directly examining the effect of COVID-19. Accordingly, follow-up studies can conduct investigations into the effect of COVID-19 on hospitality students to facilitate an understanding of the effects of COVID-19 on them.

## Data availability statement

The raw data supporting the conclusions of this article will be made available by the authors, without undue reservation.

## Ethics statement

Ethical review and approval was not required for the study on human participants in accordance with the local legislation and institutional requirements. The patients/participants provided their written informed consent to participate in this study.

## Author contributions

WL was responsible for suggesting revision to the concept and writing style of the manuscript. Y-CC was responsible for the conceptualization, investigation, methodology, and writing analyzing data for this manuscript. Both authors have read and agreed to the published version of the manuscript.
